# Cerebral Surgery

**Published:** 1905-06-24

**Authors:** 


### CEREBRAL SURGERY.

**Nerves.**—We seldom meet with reports of successful cases of divided nerves, with extensive intervals between the ends whereby direct suture is possible, treated by the various methods in vogue for bridging over the gaps. Charles A. Powers [1] contributes an article on the subject. He finds that a large proportion of cases are reported before sufficient time has elapsed to judge of the final results. Later reports of the cases do not often appear. Much of the work done is concerned with experimental operations on animals. The results in the human subject, judged by the cases gathered from literature, are disappointing. Grafting, generally of a piece of nerve from an animal, is the most popular method. He analyses the results in 22 cases of grafting which he has collected. In five only of these was any real improvement obtained. 1. At the end of two years: Sensation, "dull," and motion, fair. 2. Motion and sensation (median) practically restored at the end of six years. 3. "Good" result at the end of two years (median): Sensation, good; hand, "lame, but useful." 4. A year and a half after operation: Sensation, nearly normal; hand used daily in work, fine movements impossible. Some atrophy of muscles. 5. At the end of eight months: Motion, fair; sensation, not noted. None of these can be claimed as unqualified successes. Nine cases were recorded too early to judge of results. Eight cases were complete failures. Next to grafting come flap operations, namely, detaching a portion of the central or peripheral (or both) ends and suturing the flaps so obtained. Eleven cases are recorded, of these four were claimed as successes, five were reported too early, and two were complete failures. Ten cases of implantation or anastomosis gave five successful results, one partial success, and four failures. He concludes that foreign grafts should be abandoned, and that for the present neuroplasty and implantation are to be preferred. James Sherren [2] speaks favourably of grafts; he does not, however, cite any cases. It is recognised that a nerve-graft does not become organised into nerve-tissue, but acts merely as a scaffold in the same way that a catgut junction does, but he believes that the nerve-tissue makes a better scaffold material than any artificial substance. In making good defects of the musculo-spiral nerve the radial may be used; division or removal of the radial nerve produces, in most cases, no results that can be detected. It is not sufficiently well recognised that division of the musculo-spiral nerve is rarely followed by anæsthesia. Many of the text-books recommend that months should elapse before operating in cases of nerve injury; he points out that recovery rarely ensues without surgical intervention, and as soon as the reaction of degeneration is obtained he considers that operation should be undertaken. When a nerve is dissected out of scar-tissue or callus it should be protected by gold-foil, otherwise it will almost certainly become again involved in scar-tissue when the wound heals. Much importance is sometimes attached to details of nerve-suture; it appears immaterial what method is adopted. Simple suture through the whole nerve gives a firmer hold and involves less manipulation than suturing the sheath; it is therefore preferable. Improvement is not to be expected under twelve months, and often a much longer time must elapse. During this interval the nutrition of the skin and muscles and freedom of the joints must be maintained by massage and passive movements. Professor Langley and Dr. Anderson [3] show from experiments they have been conducting that union does not take place between the fibres of a motor united to a sensory nerve, but motor fibres will unite with motor, and sensory with sensory fibres. When union takes place in a mixed nerve it appears that some chemio-tactic influence is excited, bringing fibres of like function into relationship with each other.

**Surgery of Paralyses.**—Jackson Clarke [4] points out the disappointing results which frequently follow simple tenotomy for talipes, when little or no care is bestowed on the after treatment. A not uncommon example is a case of paralytic talipes equinus, the tendo Achillis is divided, after a few weeks the patient is allowed to walk, and before long the deformity is transformed into a talipes calcaneus, due to the excessive retraction of the calf muscles. A slight equinus in a wasted and shortened limb is often a distinct advantage, and a patient can often be much more benefited by a properly built boot than by any surgical operation.

Many cases of severe deformity from contractures may be much relieved; as an example he narrates the case of a girl with severe flexion-contractures of both hips, and complete paralysis of the extensors of both knees. The patient could progress only by grasping the feet with her hands, the thighs being flexed in contact with the abdomen, and the trunk horizontal with the ground. By a free subcutaneous division of all contracted parts the limbs were straightened, and by a suitable apparatus the child was enabled to progress in anthropoid fashion. The writer seldom performs simple subcutaneous division of the tendo Achillis; in his experience tendon-elongation gives better results. If sufficient length cannot be obtained the use of artificial silk tendons prove satisfactory. Transplantation of tendons is often of service, a healthy muscle being thus made to perform the work of a paralysed one. As an illustration of this he quotes the case of a man, aged 27, with severe flexions of both knees of 20 years' duration. The quadriceps muscles were paralysed. After free division of the contracted hamstrings and other tissues behind the knee the biceps and semi-tendinosus tendons were implanted by means of thick floss-silk into the ligamentum patellæ. Five weeks later the man could voluntarily extend his legs. Some encouraging results have been obtained in cases of obstetrical palsy of the Erb-Duchenne type. Marked improvement has followed suture of the divided or injured roots of the fifth and sixth cervical nerves. More experience, however, is required before definite statements can be made as to the value of this procedure. Tubby[5] has had a good result by muscle grafting in a case of paralysis of the serratus magnus, the result of infantile paralysis. He freed part of the sternal portion of the pectoralis major, divided it, and sutured the cut end into the cut end of the serratus magnus. Six weeks after the operation the child had gained the power of thrusting the arm forward, as in boxing, and the extended arm could be abducted well above the horizontal position. No loss of power followed the division of part of the pectoralis major.

[1] Annals of Surgery, Nov. 1904. [2] Clin. Jour., Aug. 1904. [3] Lancet, Dec. 31, 1904. [4] Pract., Sept. 1904. [5] Brit. Med. Jour., Oct. 29, 1904.

